# An Experimental Study on Mechanical Properties for the Static and Dynamic Compression of Concrete Eroded by Sulfate Solution

**DOI:** 10.3390/ma14185387

**Published:** 2021-09-17

**Authors:** Ao Yao, Jinyu Xu, Wei Xia

**Affiliations:** 1 School of Aeronautical Engineering, Air Force Engineering University, Xi’an 710038, China; xujinyuafeua@163.com (J.X.); xiaweiafeu@163.com (W.X.); 2 College of Mechanics and Civil Architecture, Northwest Polytechnic University, Xi’an 710072, China

**Keywords:** plain concrete, sulfate erosion, dynamic mechanical properties, acoustic wave testing, scanning electron microscope (SEM)

## Abstract

The mechanical properties of the static and dynamic compression of concrete eroded by a 15% sodium sulfate solution were explored with a 70-mm-diameter true triaxial static-dynamic comprehensive loading test system, and an analysis of the weakening mechanisms for the degree of macroscopic damage and microscopic surface changes of eroded concrete were conducted in combination with damage testing based on relevant acoustic characteristics and SEM scanning. The experience obtained in this paper is used to analyze and solve the problem that the bearing capacity of concrete buildings is weakened due to the decrease in durability under the special conditions of sulfate erosion. The results showed that, in a short time, the properties of concrete corroded by sulfate solution were improved to a certain extent due to continuous hydration. When the corrosion time was prolonged, the internal concrete structure was destroyed after it was eroded by sulfate, and its dynamic and static strength, deformability, and energy absorption were reduced to differing degrees, thus greatly inhibiting the overall mechanical performance of concrete; the dynamic compressive strength changed with strain that exhibited a significant strain rate effect; and, under the influence of sulfate erosion and hydration, the longitudinal wave velocity increased first and then decreased. The longitudinal wave velocity was slower than that of concrete under normal environment and distilled water immersion condition. SEM and acoustic wave analysis indicated that the internal concrete structure was destroyed after it was eroded by sulfate, and its dynamic and static strength, deformability, and energy absorption were reduced to differing degrees, thus greatly inhibiting the overall mechanical performance of concrete.

## 1. Introduction

### 1.1. Research Background

Concrete is universally used in both industrial and civil construction as a building material due to its low cost, convenience, excellent compressive performance, and easy access to components. Under normal service conditions, concrete possesses good dynamic properties; its dynamic compressive strength [1,2], deformation capacity [3,4], and energy absorption capacity [5] all show the good characteristics of building materials. However, buildings located in coastal areas [6,7] and saline-alkali areas are affected by various factors, such as saline solution and steam, for a long time under special circumstances due to their unique geographical location or environment, among which sulfate erosion is one of the more common erosion factors [8,9]. Within these conditions, sulfate erosion will compromise several of concrete’s mechanical indices and, thus, for relevant construction projects and marine works, the durability of the overall structure will be reduced due to the sulfate eroding concrete, leading to a weakened concrete that threatens lives and property.

The erosion of concrete by sulfate is mainly reflected in its destruction of the calcium silicate hydrate gel, which leads to the disruption of the original internal structural balance of concrete. Sulfate erosion usually occurs in the form of swelling and cracks in concrete [10], when after cracking, carry sulfate solution easier penetration into concrete internal pore [11], sulfate erosion in strength and durability of the concrete after key performance are affected. Under these conditions, sulfate erosion can compromise some mechanical properties of concrete [12,13]. Therefore, for related construction works and marine engineering, the durability of the overall structure will be reduced by sulfate erosion of concrete, resulting in the weakening of concrete and endangering life and property.

By now, great progresses have been made while researching the erosion effect of sulfate on concrete. In their study, Guo et al. [14,15] focused on the effects of sulfate-eroding and -weakening cementitious materials, including fiber-reinforced concrete, cement mortar, and mineral composites. Tan et al. [16,17] analyzed the performance of test pieces in the forms of concrete and concrete columns under static load and performed a compression test on the test pieces to explore relevant mechanical properties, e.g., durability.

Sarkar et al. [18,19,20,21] focused on establishing a numerical analytical simulation as well as an ion diffusion mode and model subjected to sulfatein their study. In addition, for the morphology and shape of fractures while under sulfate attack, and the changes in the external environment by sulfate from various perspectives under complicated conditions, some scholars conducted corresponding studies on specific features [22,23,24,25]. However, in some situations, some concrete in service will inevitably be affected by dynamic loads, e.g., vibrations, impacts, and explosions, which are crucial for analytical studies on the dynamic mechanical properties of concrete subjected to sulfate. In most current analytical methods for studies on the dynamic effects of sulfate on concrete, SEM or XRD [26,27] was used for analysis and interpretation from a microscopic aspect, or existing damage detection methods, such as acoustic emissions, are used for relevant analyses [28,29].

### 1.2. Research Significance

Many scholars have carried out a lot of research and analyzed sulfate erosion of concrete from many angles, including the establishment of models for different types of modified concrete and other types of cementing materials. In fact, however, most of the concrete subjected to sulfate attack is plain concrete, which has been extensively studied under the condition of erosion, but many remain static. Under actual working conditions, some concrete buildings not only bear static loads, but also dynamic mechanical properties of eroded concrete should be considered due to the influence of earthquake, war and other factors. In terms of experimental microscopic and macroscopic observation methods, it is impossible to comprehensively study the mechanism of sulfate erosion of concrete by fine observation of concrete damage points or comprehensive study of concrete damage as a whole. Therefore, dynamic and static mechanical properties are analyzed at the same time, and SEM and ultrasonic detection technology are used at the same time, so as to comprehensively meet the problems of applying different loads and analyzing different layers of sulfate corroded concrete. Sulfate corrosion of concrete for civil construction and national defense construction to provide the theoretical support and scientific basis, and to solve concrete buildings in coastal areas and saline soil areas, led to a short use period because of its special service environment, durability, and cause economic loss. By reducing the erosion of salt for concrete and improving the service time of concrete, it avoids the interference to the normal work of sulfate corroded concrete, reduces the waste of concrete caused by repeated construction of damaged parts due to erosion, and makes a small contribution to the sustainable development and reduction in carbon dioxide emissions to promote green and low-carbon construction mode.

### 1.3. Research Program

In this study, ordinary concrete cube specimens were prepared and corroded by sulfate for 0 d, 30 d, 60 d, 90 d, and 120 d, respectively; a 70-mm-diameter true triaxial static-dynamic comprehensive loading test system was utilized for mechanical testing under static and dynamic compression to obtain the dynamic stress–strain relationship for each test piece at different strain rates; and a comprehensive analysis on the damage points was conducted macroscopically by analyzing the static compressive strength, dynamic compressive strength, and strain rate effect, as well as exploring the dynamic and static mechanical properties and acoustic damage law. In addition, the corresponding damage points were scanned by SEM, so that the dynamic and static mechanical properties of concrete under sulfate attack and relevant mechanisms of action from both macroscopic and microscopic aspects may be obtained.

## 2. Materials and Methods

### 2.1. Raw Materials

**Figure 1:**
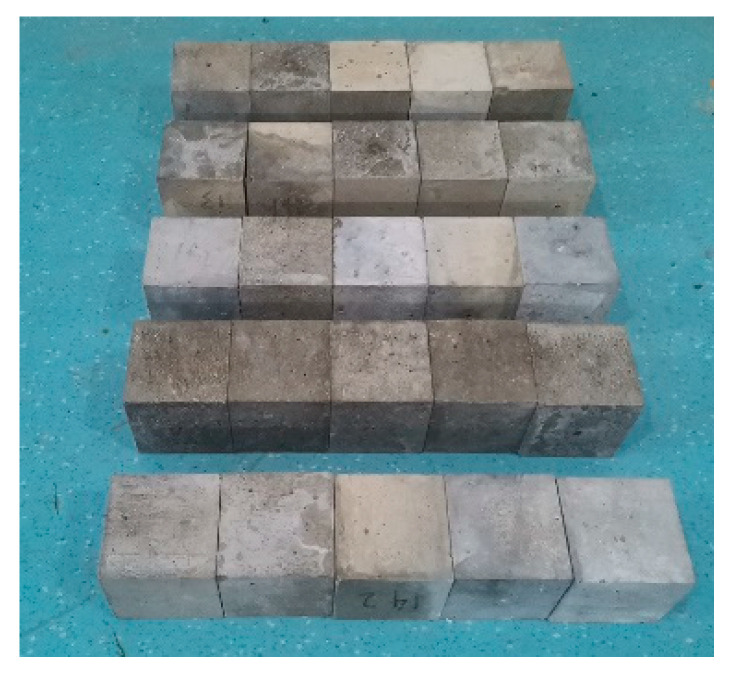
Concrete specimens eroded at different times.

For this test, Portland cement of 42.5 R P·O was one of the materials used to prepare the concrete test pieces, manufactured by Qinling Cement Group, Tongchuan, China, specific surface area: 650 m^2^/kg, density: 3.15 g/cm^3^, fineness: (80 μm sieving residue) 7.6%; fine aggregate: sand from the Ba River, Xi’an, China, fineness modulus: 2.6, apparent density: 2.44 g/cm^3^, bulk density: 1.77 g/cm^3^; coarse aggregate: crushed limestone, 15% of particle size 5–10 mm and 85% of particle size 10–20 mm, volume weight: 2700 kg/m^3^. Cement content is 495 kg/m^3^, coarse aggregate content is 1008 kg/m^3^, fine aggregate content is kg/m^3^, and water consumption is 180 kg/m^3^. A “sand enclosed cement” method was adopted for the concrete test pieces in this test. After the concrete was mixed, its surface was covered with a film to cure it and prevent water evaporation. A cube specimen with a side length of 70 mm was prepared at room temperature (80 ± 5% in case humidity and 20 ± 3 degrees in case of temperature). After 24 h of curing, the molds were removed, and the test pieces were transferred to the standard curing room for 28 d of curing. Then, the test pieces were taken out for the water to grind the sides with a stone grinding machine, so that the surfaces of the test pieces were flat and smooth. For the Na_2_SO_4_ adopted in this test, at room temperature (20 ± 3 °C), the solubility in 100 g water is about 19.5 g and the mass fraction is about 16.3%. Therefore, the erosion solution used in this test was a Na_2_SO_4_ solution with a mass fraction of 15%, and the solution was replaced every seven days, so that the mass fraction of the solution was maintained at 15% during erosion (as shown in Figure 1).

### 2.2. Test Equipment and Method

#### 2.2.1. Dynamic Compression and Static Compression Test

**Figure 2:**
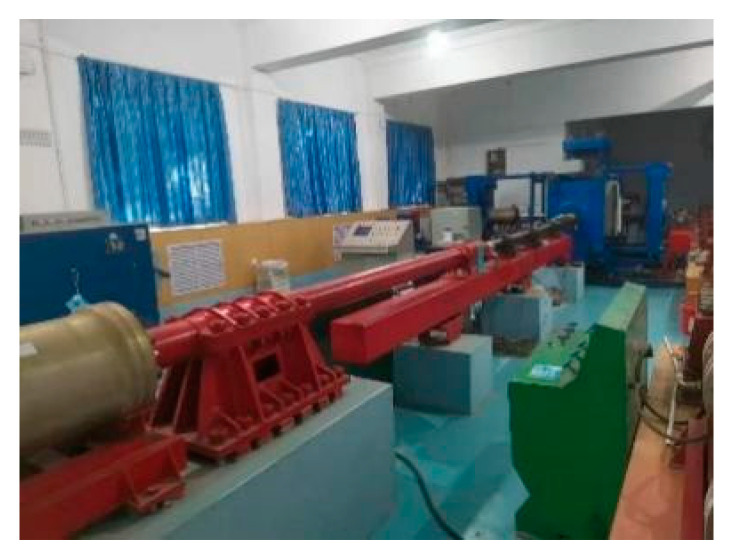
Triaxial static-dynamic comprehensive loading test system.

**Figure 3:**
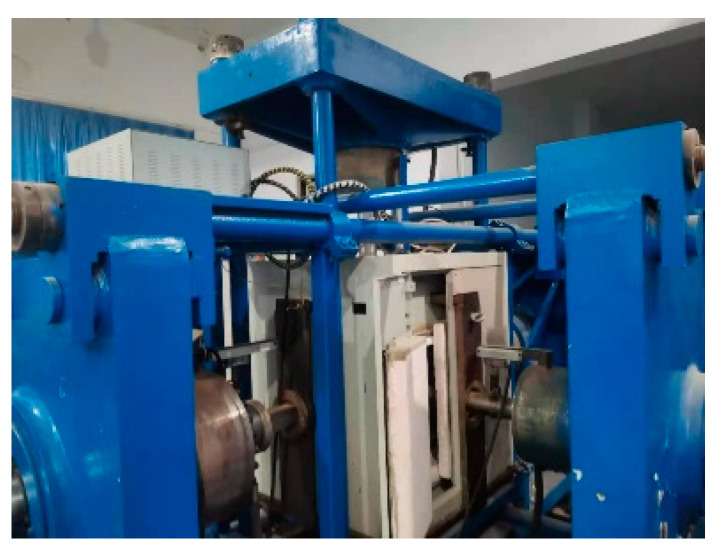
Dynamic loading system.

**Figure 4:**
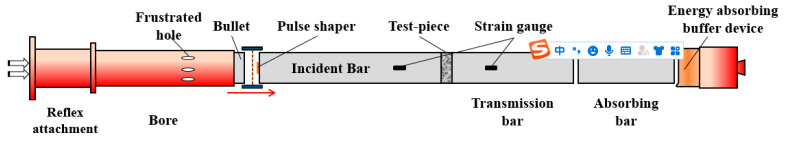
Schematic diagram for the dynamic test system components.

A self-developed true triaxial static-dynamic comprehensive loading test system (as shown in Figure 2) was adopted for this study. The system comprised of two parts. (1) The dynamic loading system (as shown in Figure 3), which is based on the split Hopkinson pressure bar (SHPB) device, and is equipped with a launching device, bullet, incident bar, transmission bar, and absorbing bar. (2) The power system, which provides power for bullets through compressed gas for dynamic tests under different high strain rates, and is comprised of an air compressor, air reservoir, and gas channel (as shown in Figure 4). The data acquisition system consists of a speed acquisition system and strain acquisition system. The static loading system is controlled by an electronic grating system and can load in six directions, achieving a true triaxial test of concrete by changing the directions of the loads and loading rates.

During static testing, the cast test pieces were divided into two groups: the S group, submerged in a 15% Na_2_SO_4_ solution; and the W group, submerged in distilled water. After submersion, both groups were tested by the true triaxial static-dynamic comprehensive loading test system. During the loading process, the load was totally in the main axis direction, i.e., there was no load in the other two directions. In the static compression test process, the loading rate of concrete samples conforms to GB 175-2007 [30]. Considering the differences in the casting and curing processes for the test pieces, for each group, the testing was in triplicate to obtain an average value, thus reducing the relevant influence from the casting process.

Before the dynamic test and to avoid the end effects caused by the friction between the end of the pressure bar and the contact surface of the test pieces, the test pieces were ground with a stone grinding machine after being soaked, and lubricant was evenly applied on both ends when testing began. During dynamic testing, the static loading device was closed with a test piece between the incident bar and the transmission bar. The launcher was started, so that the high-pressure gas generated by the air reservoir pushed the bullet to strike the incident bar at a high speed coaxially, thus producing a compressive stress pulse, which was transmitted to the test piece along the incident bar. There were two hypotheses being tested, i.e., a one-dimensional stress wave in the bar and uniform propagation of stress wave in the experimental process. The pulse information was measured by the resistance strain gauge attached to the pressure bar, and the collected test data were solved by the three-wave method to obtain the strain, strain rate, and the stress of the specimen.

(1) $$\{\begin{array}{c} \overset{•}{\varepsilon }(t)=\frac{c}{L_{s}}\left[\varepsilon_{1}(t)-\varepsilon_{R}(t)-\varepsilon_{T}(t)\right]\\ \varepsilon (T)=\frac{c}{L_{s}}\int_{0}^{T}\left[\varepsilon_{1}(t)-\varepsilon_{R}(t)-\varepsilon_{T}(t)\right]dt\\ \sigma (t)=\frac{A}{{2A}_{s}}E\left[\varepsilon_{1}(t)+\varepsilon_{R}(t)+\varepsilon_{T}(t)\right] \end{array}$$

*E* represents the young’s modulus of the bar; *c*, the wave velocity in the bar; *A*, *A_S_*, the cross-sectional areas of the bar and the test piece, respectively; *L_s_*, the initial thickness of the test piece, and *ε_I_* (*t*), *ε_R_* (*t*), and *ε_T_* (*t*), the incidence, reflection, and transmission strains in the bar, respectively. During testing, a dispersion effect may occur while propagating a stress pulse, and, furthermore, the test cannot guarantee that the test pieces have enough time to achieve a homogeneous state of stress. In this study, the loading waveform was changed by incident wave shaping technology, and the rectangular wave generated during the experiment was transformed to create a semi-sine wave that was easy to observe and utilize, thus reducing the test waveform disorder caused by the dispersion effect, and improving the overshot and high frequency oscillation in the bar impact process. During this test, a brass pulse shaper was used to shape the incident wave. This pulse shaper had a thickness of 1 mm and a diameter of 27 mm.

#### 2.2.2. Ultrasonic Test

To explore the damage law on concrete eroded by sodium sulfate, ultrasonic pulse technology was utilized for a relevant analysis on the concrete. After the sulfate attack, the concrete underwent a series of complex physical and chemical changes, with erosion products in it and on its surface, as well developing damage defects, such as pores and micro-cracks, and the overall performance of concrete was degraded due to erosion. When the ultrasonic waves passed through the concrete, longitudinal waves traveled much more quickly than shear and surface waves. Non-metallic ultrasonic analyzer is mainly composed of two parts. One part is ultrasonic transmitter, which is mainly composed of a DC boost system, a high-voltage pulse transmission system, and a transmission transducer. The other part is the ultrasonic receiving device, which is mainly composed of data acquisition system, signal amplification system, and receiving transducer. The transducer for an ultrasonic analyzer can emit various waves. Because the longitudinal wave travels most quickly, the receiving transducer receives it first, known as the head wave, of which the propagation velocity will be affected by the internal structure, pore distribution, and crack development of concrete. Therefore, the ultrasonic test of concrete corroded by sulfate mainly explores the propagation law of longitudinal wave in concrete. In this test, a KON-NM-4A nonmetallic ultrasonic analyzer manufactured by Beijing Kangkerai Engineering Testing Technology Co., Ltd., Beijing, China. was adopted for detecting acoustic damage in concrete. Before the test, in order to prevent the residual moisture on the surface of the concrete specimen from adversely affecting the test, the concrete specimen was put in a static position until there was no moisture on its surface. The treated specimen between the ultrasonic transmitting device and receiving device was fixed, the face of the concrete was fixed, preparation was completed, and ultrasonic was launched. There was then a wait for the ultrasonic wave velocity stability after the test results were recorded, and, after the record, the location of the ultrasound transmitting device and receiving device was swapped. The above steps were repeated and the ultrasonic wave velocity measurement was recorded.

#### 2.2.3. Microscopic Test

The SEM test was conducted using Nova NanoSEM230 field emission scanning electron microscope produced by FEI Company (Hillsboro, OR, USA). According to the research content and the requirements of the test instrument, the concrete sample used for observation was the concrete cement mortar part inside the square specimen damaged after static compression test. The test sample in alcohol concentration for 48 hours soaked in 70% alcohol solution was observed, and soaking by alcohol solution was performed to remove surface impurities affect the follow-up observations to improve the electrical conductivity of the sample surface, and to facilitate follow-up observations, using Leica in Germany production SCD500 type high vacuum coating apparatus coating on concrete specimens.

## 3. Results

From 0 d to 120 d, the uniaxial static compression test results of concrete specimens corroded by sulfate are as follows. From 0 d to 120 d, the compressive strength of the W group is 56.84 MPa, 62.04 MPa, 63.16 MPa, 64.98 MPa, and 65.16 MPa, respectively. The compressive strength of the S group was 56.48 Mpa, 63.14 Mpa, 62.36 Mpa, 60.65 Mpa, and 55.85 Mpa, respectively. For dynamic test results, see Table 1.

### 3.1. Dynamic Failure Mode

**Figure 5:**
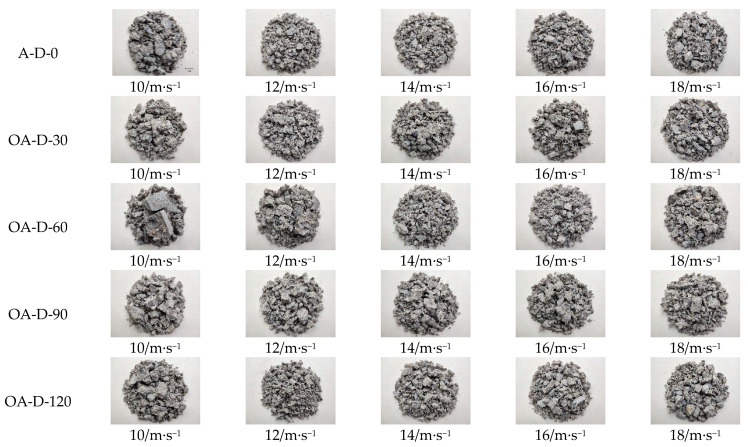
Impact failure modes of test pieces at different velocities.

The test pieces were damaged to different degrees under dynamic compression. Figure 5 shows the failure modes of the test pieces under different impact velocities Figure 5. The above failure mode diagram is scaled at a scale of 1:20 according to the actual size. At 0 d of immersion time, the failure mode was block failure with a small number of fragments, but the bulk was relatively large for the fragments, and broken coarse aggregate appeared; at 30 d of immersion time, the fragments were more integrated than at 0 d, and the bulk of the fragments was clearly larger than at 0 d; at 90 d to 120 d of immersion time, the bulk of fragments from test pieces under dynamic load decreased, and the amount of debris increased. With the increase in impact velocity and a continuous increase in strain rate, the degree of damage to the test pieces increased, and the large fragments containing coarse aggregate at low velocities were gradually split into smaller blocks until the test pieces were broken into multiple fragments, and the amount of powdery substance gradually increased with the strain rate.

### 3.2. Dynamic Stress–Strain Curve

**Figure 6:**
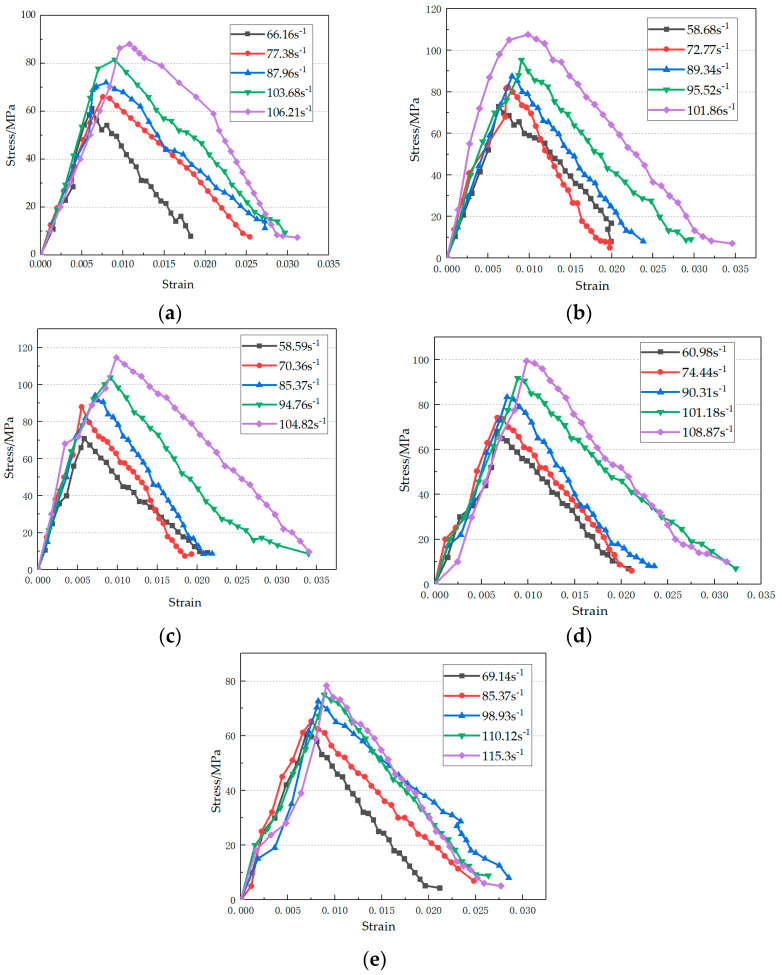
Stress–strain curve under dynamic load (**a**) OA-D-0; (**b**) OA-D-30; (**c**) OA-D-60; (**d**) OA-D-90; (**e**) OA-D-120.

The stress–strain curve may reflect a complete process from loading to failure macroscopically. Based on the “three-wave method”, the stress–strain curves for plain concrete test pieces at different strain rates and different erosion times were obtained, as shown in Figure 6. Overall, the groups of test pieces were generally the same for geometric shapes at the rising section before the peak of the stress–strain curve, and the overall trend was an inverted “V” shape. As strain increased, stress initially increased, then decreased. The process can be roughly divided into three stages: compaction, linear elastic deformation, and yield failure. The reason behind this was that, for the concrete test pieces under impact load, the original pores formed by air bubbles during casting were first squeezed and compacted, and at this point, strength slowly increased. With the continuous increase in strain rate, the test pieces entered the linear elastic deformation stage, within which an approximate linear relationship between stress growth and deformation manifested. When the yield point was reached, the test pieces were further damaged, and the stress increased slowly again. When the load peaked, the test pieces were destroyed with residual strength having gradually decreased. In the rising stage of the stress–strain curve, the rising trend of the specimen was roughly the same at different impact velocities. With the increasing impact velocities, the height of the highest point of the stress–strain curve also increased. At the same time, the end point of the stress–strain curve moved roughly to the right with the increase in impact velocity. After the erosion, for the four test piece groups, i.e., OA-D-30, OA-D-60, OA-D-90, OA-120-D, the dynamic stress–strain curve was significantly influenced by the strain rate.

### 3.3. Dynamic Mechanical Related Properties

The dynamic compressive strength of the test pieces depended on the stress value corresponding to the peak point of the stress–strain curve, reflecting the characteristics of concrete in strength under an impact load.

The dynamic strength growth factor ($I_{c}^{d}$) is defined:

(2) $$I_{c}^{d}={f_{c,d}/f}_{c,s}$$

where $f_{c,s}$, $f_{c,d}$ represent the compressive strengths of concrete under static and dynamic loads, respectively.

**Figure 7:**
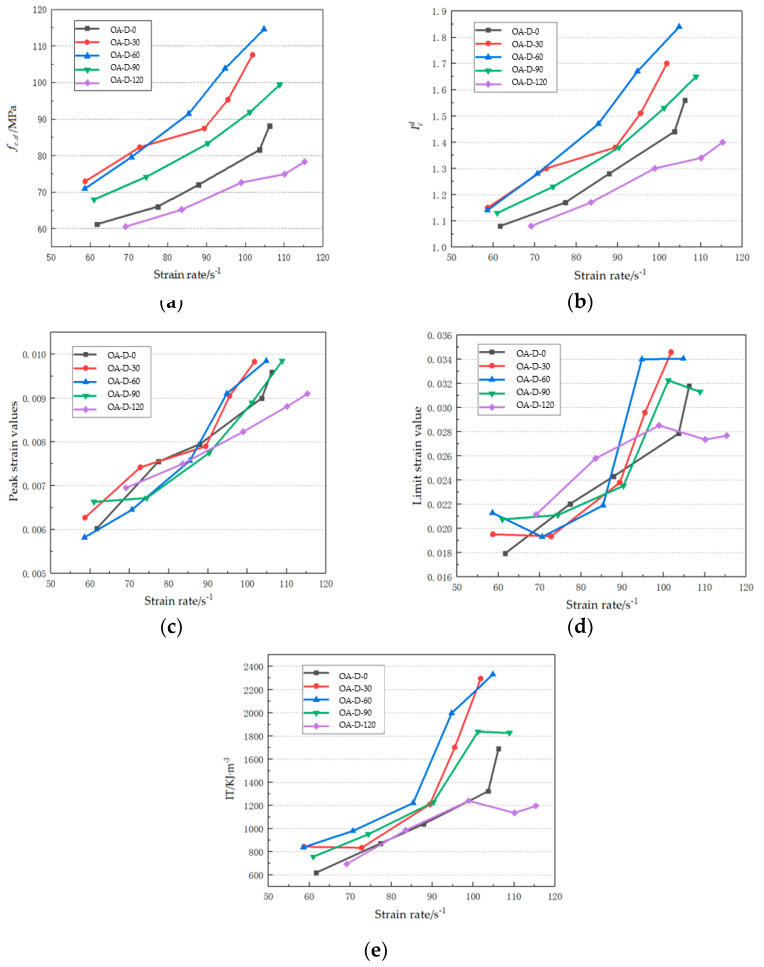
Dynamic mechanical related properties (**a**) dynamic compressive strengths of concrete; (**b**) dynamic strength growth factors; (**c**) peak strain; (**d**) limit strain; (**e**) impact toughness.

$I_{c}^{d}$ represents the ratio of dynamic compressive strength to static compressive strength. Figure 6 shows the dynamic growth factors of test pieces at different strain rates. Obviously, for each test piece, the $I_{c}^{d}$ value is greater than 1, indicating that for all test pieces, the dynamic compressive strength is greater than the static compressive strength. These circumstances can be explained based on the fundamental principles governing concrete failure: for a concrete test piece under an external load, there must be stress deformation, and at this point, one or more major cracks spread first due to the poor ductility of concrete, thus reducing the probability of smaller cracks to form. Under static load, it takes a long time for major cracks to form and spread, and finally, the cracks may pass through the test piece and cause it to break it into fragments; under high-speed impacts, the loading rate of the dynamic load accelerates, and the cracks in concrete cannot completely react during the deformation process, i.e., there will be some lag, and the destruction of concrete is not entirely caused by main cracks developing. Due to the extremely high speed, the main cracks have no time to develop through the weak areas, but tend to spread out in a straight line, passing through the concrete matrix and aggregate, and absorbing more energy with a large number of micro-cracks. The dynamic strength growth factor represents the increase in amplitude of the compressive strength of a test piece under an impact load relative to that under static load, reflecting the enhancement effect of dynamic loads on concrete strength. Figure 7 shows the relationship between strain rate and dynamic compressive strength. (1) After sulfate erosion, a significant positive correlation existed between dynamic compressive strength and strain rate for the five test piece groups, and the dynamic compressive strength increased linearly with the strain rate. (2) At the same strain rate, the dynamic compressive strengths of the two test pieces groups eroded for 90 d and 120 d were weaker than that of the two groups eroded for 0 d and 30 d, indicating that Na_2_SO_4_ erosion decreases the concrete dynamic load bearing capacity to a certain extent, and a longer submersion time leads to greater damage to the dynamic compressive strength of concrete itself. (3) For the two test piece groups eroded for 90 d and 120 d, and with the increase in strain rate, the growth rate of dynamic compressive strength decreased gradually, indicating that, at the same strain rate, for the test piece groups with longer submersion times in the Na_2_SO_4_ solution, the dynamic compressive strength loss increased with the strain rate. (4) The dynamic strength growth factor also had an obvious enhancement effect on the strain rate, and the effect of the dynamic strength growth factors in the test groups with longer submersion times were weaker than in the test groups with shorter submersion times.

In this study, the impact toughness (*IT*) represents the energy change in concrete under dynamic load, i.e., energy absorption capacity. Physically, it is the area under the stress–strain curve, which may be expressed as:

(3) $$IT=\int_{0}^{\varepsilon_{u}}fd\varepsilon$$

where *f* represents the dynamic stress–strain curve of the test piece and *ε_u,_* is the dynamic limit strain of the curve. Dynamic compressive deformation is an important reference index for analyzing mechanical characteristics of concrete under impact loads after sulfate attacks, with the peak strain (*ε_p_*) and limit strain (*ε_u_*) acting as the important mechanical parameters for the impact compressive deformation performance of concrete. Under different strain rates, impact toughness, peak strain, and limit strain have roughly the same upward trend, as shown in Figure 7. These three dynamic mechanical properties all increased with the increase in strain rate. In the initial stage of sulfate solution erosion, the concrete of OA-D-30 and OA-D-60 test groups, in particular, had generally superior dynamic properties in this test. The dynamic mechanical properties of the oA-D-0 group were in the middle position between the early and late stage of erosion. The erosion time exceeds 60 d when the dynamic mechanical properties of OA-D-60, OA-D-90, and OA-D-120 are in a declining trend, and when the performance of OA-D-90 and OA-D-120 are generally lower than that of OA-D-0.

## 4. Analysis of Relevant Mechanisms

After the sulfate attack, the concrete underwent a series of complex physical and chemical changes. The internal defects of concrete are detected by using the principle of different transmission speed of ultrasonic wave in different media. Therefore, this test is for studying the law of longitudinal wave propagation in concrete after undergoing a sulfate attack.

**Figure 8:**
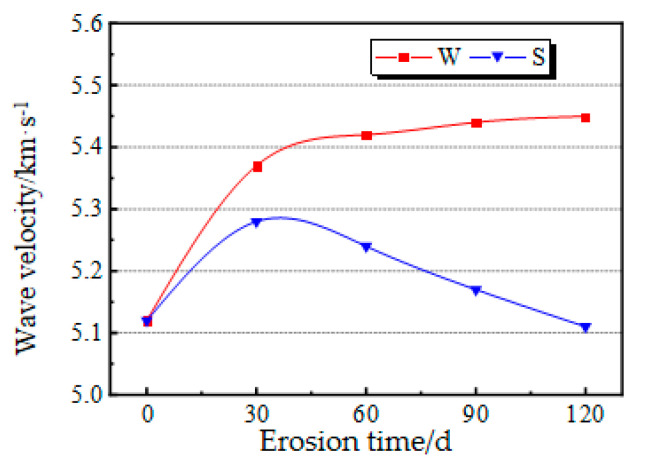
Changes in wave velocity with erosion time.

The experimental results of ultrasonic wave velocity of concrete after sulfate erosion are as follows: from 0 d to 120 d, the ultrasonic wave velocity of the W group is 5.12 km/s, 5.37 km/s, 5.42 km/s, 5.44 km/s, and 5.45 km/s, respectively. The ultrasonic wave velocities of the S group were 5.12 km/s, 5.28 km/s, 5.24 km/s, 5.17 km/s, and 5.11 km/s, and the relationship between the longitudinal wave velocity obtained by ultrasonic testing with erosion time is shown in Figure 8. Obviously, for the test pieces, the increasing trend of longitudinal wave velocity with erosion time was like that of the static compressive strength. The transmission speed of ultrasonic wave in concrete reflects the structural strength and integrity of concrete itself. Usually, the wave speed is fast and the structure is intact and not destroyed, while the wave speed is slow when it is eroded. The wave velocity of the W group rose continuously, and the amplitude within 30 d after immersion was at its maximum, i.e., 76% of the total growth rate. In the W group, with the increase in soaking time, the wave velocity of concrete in pure water increased due to continuous hydration. The longitudinal wave velocity of the S group first increased and then decreased, peaking 30 d after submersion, and decreased continuously after then. At 60 d after submersion, the longitudinal wave velocity decreased to about the initial value, which was 93.76% of the wave velocity of the W group. In the S group, due to the dual influence of hydration of concrete and sulfate erosion, hydration played a dominant role in the 0 d and 30 d tests, so the wave velocity increased. When the erosion time exceeded 30 d, the sulfate erosion intensified, and the negative effect of concrete soaked in solution was greater than the positive effect of hydration, so the wave velocity decreased.

**Figure 9:**
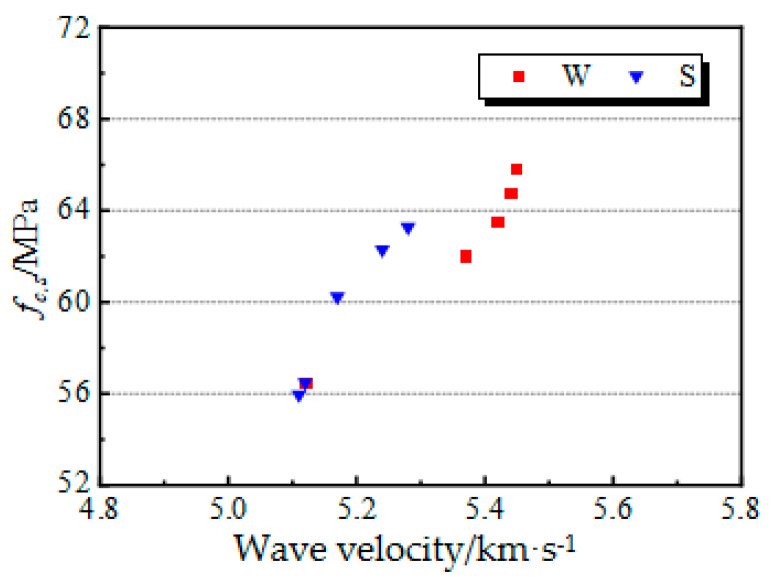
Relationship between longitudinal wave velocity and static compressive strength.

Many scholars studied the compressive strength, elastic modulus, and the relationship between the dynamic elastic modulus and ultrasonic velocity for concrete. Overall, the fitting relationship between static compressive strength and ultrasonic velocity was good. To analyze the relationship between the ultrasonic velocity and the damage degree for the test pieces in this study, a regression analysis of ultrasonic velocity and concrete compressive strength was conducted using Formulas (4) and (5), respectively, with the static compressive strength as a criterion for damage degree.

(4) $$f_{c,s}=a{v_{p}}^{b}$$

(5) $$f_{c,s}=ae^{bv_{p}}$$

where *a* and *b* represent empirical coefficients. Figure 9 shows the relationship between the longitudinal wave velocity and static compressive strength of the test pieces.

**Figure 10:**
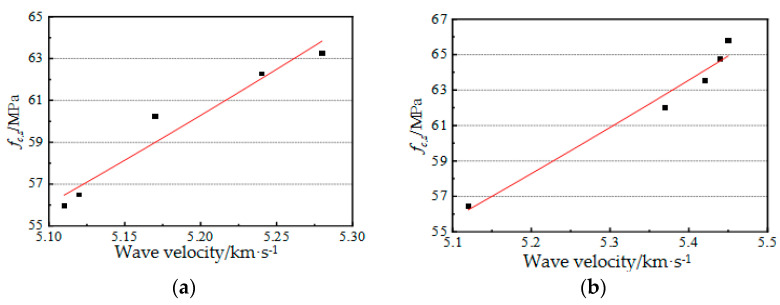
Fitting curve of the static compressive strength and wave velocity for the test pieces (**a**) the W group; (**b**) the S group.

The fitting results are shown in (6), and the relevant relation diagram is shown in Figure 10. The fitted correlation coefficient R^2^ = 0.9679 (the W group), R^2^ = 0.9471 (the S group). Thus, the correlation was high, and the fitting results were optimal.

(6) $$\left\{ \begin{array}{l} W:f_{c,s}=1.316{v_{p}}^{2.299}\;(R^{2}=0.9679)\\ S:f_{c,s}=0.123{v_{p}}^{3.748}\;(R^{2}=0.9471) \end{array}\right.$$

The compressive strength and erosion resistance coefficient were used to analyze the degradation law of concrete test pieces. Therefore, according to classical mechanics, D, the damage variable, is defined by the elastic modulus of material as such:

(7) $$D=1-\frac{\tilde{E}}{E}$$

where E—the elastic modulus of material in its initial state;

$\tilde{E}$—the elasticity modulus of material after damage

The following can be obtained according to the Formula (8):

(8) $$E={v_{p}}^{2}\rho \frac{\left(1+\mu \right)\left(1-2\mu \right)}{1-\mu }$$

Substituting it into the Formula (8), the test piece damage variable defined by the wave velocity can be obtained as:

(9) $$D=1-{\left(\frac{{\tilde{v}}_{p}}{v_{p}}\right)}^{2}$$

**Figure 11:**
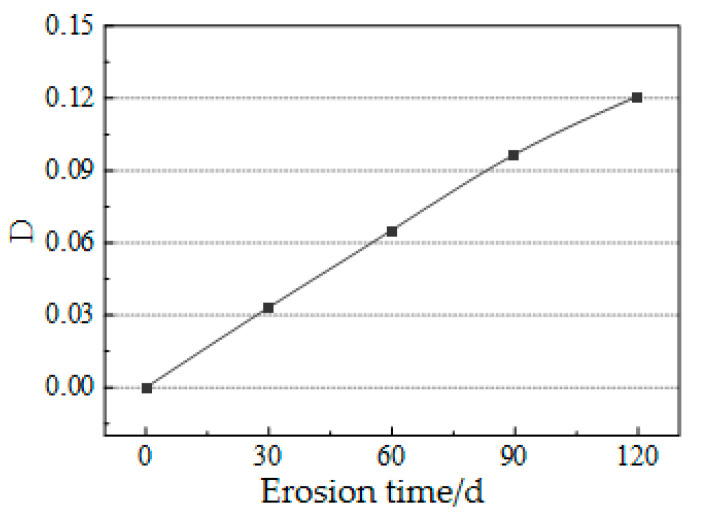
Damage variable of test pieces after sulfate attacks.

Concrete specimens are affected by the erosion of sodium sulfate solution, which mainly includes the erosion of sulfate and the continuous hydration of concrete. In order to separately analyze the influence of sulfate erosion on concrete, where ${\tilde{v}}_{p}$—the wave velocity of material in its initial state, and $v_{p}$—the wave velocity of material after damage. According to the Formula (9), the damage degree of material before any damage was 0, and the damage degree of material after any damage was 1. The damage curve of the test pieces (as shown in Figure 11) was drawn. Obviously, the damage degree of the concrete test pieces after undergoing sulfate attacks continuously increased with erosion time. When the erosion time was 120 d, the damage degree was up to 0.12.

**Figure 12:**
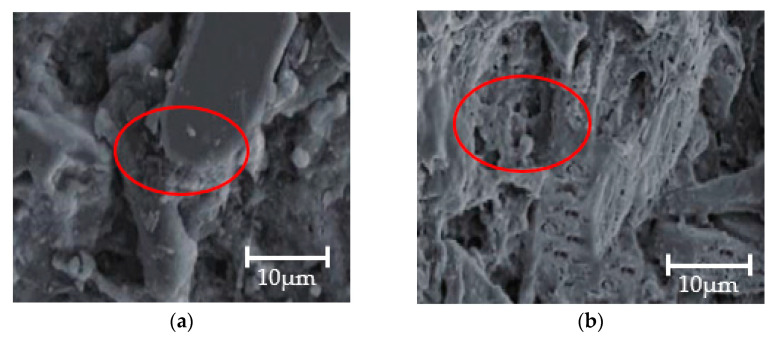
Microstructure of concrete after soaking (**a**) W group; (**b**) S group.

The acoustic velocity of the W group soaked in distilled water is the initial speed of the material, while that of the S subjected to sodium sulfate erosion is the speed after the material was damaged. The static mechanical properties and dynamic mechanical properties decreased to different degrees as the wave velocity decreased based on acoustic measurements. Figure 12 shows the microscopic morphology of specimens of the W group and the S group, respectively, at a magnification of 2000 times. It can be seen from the figure that, after the resoaking time reaches 120 d, a large amount of consolidation products of Na_2_SO_4_ occurred between the skeleton and the pore bulk, and there were many consolidation products in the pores and increasing consolidation products amplify the defects. The static mechanical properties and dynamic mechanical properties decreased to different degrees as the wave velocity decreased based on acoustic measurements.

## 5. Discussion

Through the tests, the static and dynamic mechanical properties measured in this study were basically consistent with the performance of concrete under the condition of erosion [31]. Through the static and dynamic tests, it is found that, in the process of sulfate solution erosion of concrete, the static and dynamic mechanical properties of concrete were improved to a small extent [32]. The performance of specimens decreased with the prolongation of erosion time. Through the method of ultrasonic detection, the specimen was tested, and the relationship between wave velocity and the damage degree of components was found to be matched with the results of dynamic and static mechanics tests in the early stage, and the changes in the concrete interior during the erosion process were further determined through microscopic observation.

Throughout the sodium sulfate erosion process, the changes in static compressive strength and dynamic compressive strength of the concrete test pieces were primarily influenced by the following factors. (1) During the process of sulfate erosion, the test pieces were submerged in the solution. During the curing process, the concrete continued to hydrate with time, and the hydration continued during the submersion process. (2) In the process of sulfate solution immersion, concrete continues to hydrate, and sulfate also begins to enter into concrete. The reason why sulfate enters into concrete is that there is no liquid in the internal pores of concrete. Solution immersion makes liquid with sulfate enter into the sample due to the influence of water pressure. In addition, because the concrete surface has more pore structure, it also promotes the sulfate solution to enter the concrete interior. (3) During the hydration process for concrete, a calcium silicate hydrate gel was formed to fill in the defects and pores in the concrete pouring process. This makes the concrete stronger, and, as sulfate attacks concrete, while the consolidation products strength was lower than that of normal calcium silicate hydrate gel, it also led to an internal force effect, for which the overall damage degree of material was increased.

In order to obtain the erosion situation of concrete buildings in service under special environment, the conclusion of ultrasonic test on concrete eroded by sulfate solution in this paper can be used to obtain the current damage state of concrete. In the actual working conditions, the concrete building may have eroded parts of ultrasonic detection, test data, and damage fitting curve comparison; thus, using the comparison of the results of the building performance and safety performance has a better evaluation and analysis. The damaged parts could bear the main dynamic load or static load of the position of reinforcement treatment to ensure that the concrete service during the work and safety, and, at the same time, to avoid a large range of reinforcement and mix anti-erosion treatment in the premise of ensuring safety to reduce the amount of concrete to achieve the purpose of low carbon environmental protection.

## 6. Conclusions

In this study, the split Hopkinson pressure bar (SHPB) experiment was conducted with a true triaxial static-dynamic comprehensive loading test system.

Static and dynamic mechanical testing of concrete was conducted 0 d, 30 d, 60 d, 90 d, and 120 d after undergoing erosion by a sodium sulfate solution with a mass fraction 15%. Acoustic waves were used for macroscopical detection and SEM for microscopical analysis. The main conclusions are:

(1) The dynamic and static mechanical properties of concrete are weakened to a certain extent after sulfate erosion. The dynamic strain rate effect is significant, the dynamic compressive strength is decreased, and the impact toughness and deformation capacity are also weakened.
(2) There are two main factors affecting the dynamic and static mechanical properties of concrete after being corroded by sulfate solution, including continuous hydration of concrete in solution and sulfate erosion. These two effects have two completely opposite influence trends on the dynamic and static mechanical properties of concrete. Hydration strengthens the properties of concrete, while sulfate erosion weakens the properties.
(3) The law of changes in longitudinal wave velocity is similar to that of strength. Due to the changes in strength and internal pore structure of the test pieces after sulfate erosion. The longitudinal wave velocity is slower than that of concrete under normal environment and distilled water immersion condition. In addition, the compressive strength and the compressional wave velocity develop. In the same direction, in response to external influences, both the compressive strength and the compressional wave velocity of concrete have a decreasing trend after sulfate erosion.
(4) For the concrete eroded by sulfate, the internal structure changed, within which a large number of low-strength consolidation products may be formed. In addition, the increase in the volume of reaction products and the crystals with strength weaker than that of normal gels may compromise the concrete strength.

## Figures and Tables

**Table 1:** Uniaxial dynamic compression test results of concrete test pieces after sulfate attack.

Serial Number	Erosion Time	Impact Speed/m·s^−1^	$\bar{\dot{\varepsilon }}/s^{-1}$	$f_{c,d}/\mathrm{MPa}$	$\varepsilon_{p}/\times 10^{-3}$	$\varepsilon_{u}/\times 10^{-3}$	$I_{c}^{d}$	*IT* (kJ/m^3^)
OA-D-0	0 d	10	61.66	61.25	6.02	17.94	1.08	619.27
		12	77.38	66.03	7.55	22.04	1.17	872.59
		14	87.96	72.04	7.95	24.32	1.28	1041.13
		16	103.68	81.59	9.00	27.86	1.44	1323.35
		18	106.21	88.11	9.59	31.77	1.56	1691.18
OA-D-30	30 d	10	58.68	72.95	6.27	19.52	1.15	843.77
		12	72.77	82.31	7.42	19.35	1.30	835.12
		14	89.43	87.46	7.90	23.81	1.38	1210.49
		16	95.52	95.32	9.05	29.60	1.51	1702.45
		18	101.86	107.56	9.83	34.59	1.70	2295.51
OA-D-60	60 d	10	58.59	70.95	5.82	21.29	1.14	839.00
		12	70.63	79.61	6.45	19.30	1.28	980.63
		14	85.37	91.41	7.57	21.91	1.47	1221.35
		16	94.76	103.86	9.10	34.00	1.67	1999.34
		18	104.82	114.61	9.85	34.06	1.84	2331.36
OA-D-90	90 d	10	60.98	68.00	6.63	20.75	1.13	757.76
		12	74.44	74.18	6.72	21.11	1.23	952.03
		14	90.31	83.34	7.75	23.53	1.38	1228.89
		16	101.18	91.86	8.89	32.26	1.53	1838.85
		18	108.87	99.38	9.85	31.31	1.65	1827.22
OA-D-120	120 d	10	69.14	60.59	6.95	21.15	1.08	694.53
		12	83.57	65.21	7.50	25.80	1.17	986.77
		14	98.93	72.62	8.23	28.53	1.30	1240.58
		16	110.12	74.94	8.81	27.36	1.34	1135.85
		18	115.30	78.34	9.10	27.68	1.40	1196.65

## Data Availability

The data used to support the findings of this study are available from the corresponding author upon reasonable request.
